# A yeast strain associated to *Anopheles* mosquitoes produces a toxin able to kill malaria parasites

**DOI:** 10.1186/s12936-015-1059-7

**Published:** 2016-01-11

**Authors:** Matteo Valzano, Valentina Cecarini, Alessia Cappelli, Aida Capone, Jovana Bozic, Massimiliano Cuccioloni, Sara Epis, Dezemona Petrelli, Mauro Angeletti, Anna Maria Eleuteri, Guido Favia, Irene Ricci

**Affiliations:** School of Biosciences and Veterinary Medicine, University of Camerino, 62032 Camerino, Italy; Department of Veterinary Sciences and Public Health, University of Milan, 20133 Milan, Italy

**Keywords:** *Wickerhamomyces anomalus*, *Plasmodium berghei*, *Anopheles stephensi*, Malaria, Symbiotic control, Killer toxin

## Abstract

**Background:**

Malaria control strategies are focusing on new approaches, such as the symbiotic control, which consists in the use of microbial symbionts to prevent parasite development in the mosquito gut and to block the transmission of the infection to humans. Several microbes, bacteria and fungi, have been proposed for malaria or other mosquito-borne diseases control strategies. Among these, the yeast *Wickerhamomyces anomalus* has been recently isolated from the gut of *Anopheles* mosquitoes, where it releases a natural antimicrobial toxin. Interestingly, many environmental strains of *W. anomalus* exert a wide anti-bacterial/fungal activity and some of these ‘killer’ yeasts are already used in industrial applications as food and feed bio-preservation agents. Since a few studies showed that *W. anomalus* killer strains have antimicrobial effects also against protozoan parasites, the possible anti-plasmodial activity of the yeast was investigated.

**Methods:**

A yeast killer toxin (KT), purified through combined chromatographic techniques from a *W. anomalus* strain isolated from the malaria vector *Anopheles stephensi*, was tested as an effector molecule to target the sporogonic stages of the rodent malaria parasite *Plasmodium berghei*, in vitro. Giemsa staining was used to detect morphological damages in zygotes/ookinetes after treatment with the KT. Furthermore, the possible mechanism of action of the KT was investigated pre-incubating the protein with castanospermine, an inhibitor of β-glucanase activity.

**Results:**

A strong anti-plasmodial effect was observed when the *P. berghei* sporogonic stages were treated with KT, obtaining an inhibition percentage up to around 90 %. Microscopy analysis revealed several ookinete alterations at morphological and structural level, suggesting the direct implication of the KT-enzymatic activity. Moreover, evidences of the reduction of KT activity upon treatment with castanospermine propose a β-glucanase-mediated activity.

**Conclusion:**

The results showed the in vitro killing efficacy of a protein produced by a mosquito strain of *W. anomalus* against malaria parasites. Further studies are required to test the KT activity against the sporogonic stages in vivo, nevertheless this work opens new perspectives for the possible use of killer strains in innovative strategies to impede the development of the malaria parasite in mosquito vectors by the means of microbial symbionts.

**Electronic supplementary material:**

The online version of this article (doi:10.1186/s12936-015-1059-7) contains supplementary material, which is available to authorized users.

## Background

Malaria is one of the most alarming infectious diseases threatening millions people, mostly in sub-Saharan regions [1, 2]. It is caused by *Plasmodium* protozoan parasites and transmitted by *Anopheles* mosquitoes. Even though several traditional remedies are in use, the disease continues to represent a heavy health burden in endemic countries. In absence of effective vaccines, public health programmes have focused their resources on the use of insecticides to reduce the number of vector populations, and/or drugs to kill directly the pathogens [3].

Malaria control programmes involving chemical and pharmacological treatments are not always sustainable due to several factors, such as economic costs and logistic aspects. In addition, both vectors and parasites have enhanced resistance against many commonly used pesticides and medicines. Confirmed resistance of 125 mosquito species lead the Malaria Eradication Research Agenda to state that novel control strategies are urgently requested for malaria suppression [4]. In this frame, a new tool called “Symbiotic Control” (SC) has been recently proposed. The SC is a multifaceted approach that exploits symbiotic microorganisms to control insect pests reducing their vector capabilities [5, 6]. This strategy implies the identification of suitable microbes able to spread among the vector populations. Several microbes have been proposed for malaria-SC, including the bacteria *Asaia*, *Wolbachia*, *Pantoea agglomerans*, *Elizabethkingia meningoseptica* and the fungi *Metarhizium robertsii* and *Wickerhamomyces anomalus* [7–12].

The yeast *W. anomalus* has been recently isolated from different mosquito species [13] and its intimate association with its host has been well characterized in the Asian malaria vector *Anopheles stephensi*, where the yeast localizes to gut and gonads, suggesting specific biological roles in these anatomical districts [12]. Similar to various environmental strains used as bio-preservation agents for their anti-bacterial/fungal activities in food industry, the *W. anomalus* strain isolated from *An. stephensi* secretes an antimicrobial KT, which might exert an antiseptic function in the mosquito [14, 15]. Previous results showed the effective antimicrobial activity of the KT, produced by *W. anomalus* strain of mosquito against other targeted yeasts [14]. The *W. anomalus* potency to be stimulated for KT production was demonstrated, whose releases in the mosquito midgut and gonads showing long-lasting features [14]. Consequently, the possible KT anti-plasmodial effect against parasite developmental stages was investigated, taking place in mosquito gut. In the present study, a strong in vitro antimicrobial activity of the KT secreted by the *W. anomalus* strain isolated from *Anopheles stephensi* against the sporogonic stages of the malaria rodent parasite *Plasmodium berghei* was determined and a killing mechanism of action based on a β-glucanase enzymatic activity was proposed.

## Methods

### Yeast strains

Three *W. anomalus* strains were used: (1) *Wa*F17.12 isolated from *An*. *stephensi* [12], (2) *Wa*ATCC 96603 a KT-producer reference strain and (3) *Wa*UM3 a *Wa*KT-non-producing and, furthermore, *Wa*KT-susceptible strain [16, 17]. These yeasts were grown in YPD liquid medium (20 g/l peptone, 20 g/l glucose, 10 g/l yeast extract) buffered at pH 4.5 with 0.1 M citric acid and 0.2 M potassium phosphate dibasic and incubated at 26 °C for 36 h at 70 rpm to stimulate the production of KTs [18, 19].

### Purification of yeast KTs

The yeast cultures obtained after 36 h incubation were centrifuged at 3000 rpm for 10 min to remove cells. The supernatants were filtered using 0.22 μm nitrocellulose membranes, and concentrated 50× using an Amicon system equipped with Millipore Ultracell ultrafiltration disk (MWCO: 10 kDa). The samples were further concentrated using Pierce Concentrators centrifugal ultrafiltration tubes with a MWCO of 9 kDa (Thermo Fisher Scientific Inc). Resulting solutions were analysed by anion-exchange chromatography on a FPLC AKTA Basic device equipped with a HiTrap DEAE FF column (GE Healthcare) using a linear gradient of buffer A (0.1 M citric acid and 0.2 M K_2_HPO_4_, pH 4.5) and buffer B (0.1 M citric acid and 0.2 M K_2_HPO_4_, pH 4.5), flow rate 5 ml/min.

Retained and non-retained fractions for each strain were collected from different runs, quantified for protein content according to the Bradford’s method [20] and tested for the presence of killing activity against the susceptible strain *Wa*UM3, as described by Cappelli [14]. Prior to anti-*P. berghei* activity assay, the non-retained fractions of *Wa*F17.12 and *Wa*ATCC 96603 showing the KT activity, and the non-retained fraction of *Wa*UM3 (negative control), were buffer-exchanged with an Hi-Trap desalting column to PBS 1× (10 mM Na_2_HPO_4_, 2.7 mM KCl, 138 mM NaCl, pH 7.4) to prevent possible interference, like pH-incompatibility, with the parasite development.

### Ethics statement

BALB/c mice were reared in the animal facilities of the University of Camerino (Italy). Experimental animal rearing and handling were fully in agreement with the Italian Directive 116 of 10/27/92 on the “use and protection of laboratory animals”, and in compliance with the European regulation (86/609) of 11/24/86 (license no. 125/94A, issued by the Italian Ministry of Health). The experimentation was approved by the Ethical Committee of University of Camerino.

### Mice infection and *Plasmodium berghei* sporogonic stages cultures

Five-week-old BALB/c mice were infected with a recombinant *P. berghei* strain (*Pb*CTRPp.GFP) from a cryopreserved stock, as previously described by Vlachou et al. [21]. This transgenic parasite expresses the Green Fluorescent Protein (GFP) during ookinete development under the control of the *ctrp* promoter, whereas no signal is exhibited in the asexual blood stages and gametocytes. After 4 days, mice parasitaemia and haematocrit were evaluated using Giemsa staining and Neubauer’s chamber, respectively. Mice with a parasitaemia in the range 5–10 % were selected as donors, and 10^7^ infected red blood cells (IRBC) were inoculated into healthy mice, previously treated with phenylhydrazine to induce reticulocytosis [22]. After further 4 days, exflagellation centres were counted as indicators of parasite infectivity [23], using an optical microscope with a 40× objective (Carl Zeiss Axio Observer.Z1, Milan, Italy). To induce exflagellation, 5 µl of infected blood were blended with 120 µl of incomplete ookinete medium (16.4 g/l RPMI1640 containing 25 mM HEPES/l-glutamine, 2 % NaHCO_3_, 0.05 % hypoxanthine, 100 µM xanthurenic acid, pH 8) and incubated 20 min at 22 °C as reported by Ghosh et al. [24]. After the check of exflagellation centers, gametocytaemic blood was collected by cardiac puncture.

*Plasmodium berghei* sporogonic stages were obtained, in vitro, culturing 20 µl of infected blood with 180 µl of complete ookinete medium in a 96-wells microtitre plates. Complete medium was prepared supplementing the above-mentioned incomplete medium with 20 % heat inactivated fetal bovine serum (Invitrogen), 50 U/ml penicillin and 50 µg/ml streptomycin (Invitrogen). Ookinetes developed after about 24 h at 19 °C.

### In vitro *Wa*KTs anti-plasmodial activity

The fractions containing the KTs obtained by chromatographic analysis of the extracts, from *Wa*F17.12, *Wa*ATCC 96603 (positive control) and *Wa*UM3 (negative control) were tested against *P. berghei* sporogonic stage cultures prepared as described above. Four concentrations (25, 60, 75, 100 µg/ml) of KTs from both *Wa*F17.12 and *Wa*ATCC 96603 were tested; whereas for *Wa*UM3, the purification product was tested at the highest concentration possible to evaluate interference due to buffer solution. Concurrently, in each control well, PBS 1× pH 7.4 (without KT) was added to ookinete complete medium and gametocytaemic blood. After 24 h incubation at 19 °C, the KTs anti-plasmodial activity was estimated comparing the number of fluorescent *P. berghei* sporogonic stages in both control and sample wells using a fluorescence microscope and 40× objective (Carl Zeiss Axio Observer.Z1, Milan, Italy). The assays were performed in triplicate using 96-wells microtitre plates and the results were reported as the averages of inhibition percentages of parasite development.

The LC_50_ (KTs concentration at which 50 % of the parasite population is killed) was evaluated after 24 h. This value was determined by nonlinear regression analysis plotting the number of sporogonic stages versus log[KT] with GraphPad Prism 5 software as described by Savoia et al. [25].

### Statistical analysis

Five runs of parasite cultures were obtained during the experimentation and the results of each run were reproducible. All the experiments against *Plasmodium* sporogonic stages were repeated in triplicate. The data obtained from parasite counts were analysed with GraphPad Prism 5 software and statistical analysis was carried out by One Way ANOVA followed by Bonferroni’s Multiple Comparison Tests. Statistical significance is expressed as a *p* value <0.05.

### Morphological analysis of *Plasmodium berghei* sporogonic stages using Giemsa staining

Control and KT-treated (KTs concentration 100 μg/ml) *P. berghei* sporogonic stages slides were investigated for possible damages induced by KT-activity on the zygote and/or ookinete morphologies. Briefly, 5 μl of parasite cultures were smeared onto a glass slide, fixed by methanol for 15 min at room temperature and stained for 45 min with 10 % Giemsa solution (Sigma-Aldrich) in PBS 1× pH 7.4. After this incubation period the samples were analysed using a microscope and 100× objective (Carl Zeiss Axio Observer.Z1, Milan, Italy).

### Castanospermine assay

Purified KTs (100 μg/ml) from both *Wa*F17.12 and *Wa*ATCC 96603 were pre-incubated with 25 µM of castanospermine (Sigma-Aldrich), a β-glucanase inhibitor, for 1 h at 25 °C under static conditions [26] and, then, added to the *P. berghei* cultures, in triplicates. The inhibitory effect of the indolizine alkaloid on the antimicrobial activity of KTs was evaluated on *P. berghei* sporogonic stage cultures after 24 h incubation at 19 °C and it was indicated as the average of three replicates. Upon castanospermine treatment, the number of parasites was compared to controls.

## Results

### Purification of KTs

The KTs from *W. anomalus* strains were purified to test their ability to inhibit the development of *P. berghei* sporogonic stages. The purification process started with the production of the KT in yeast culture medium, and proceeded with a series of concentrations and chromatographic steps.

**Fig. 1:**
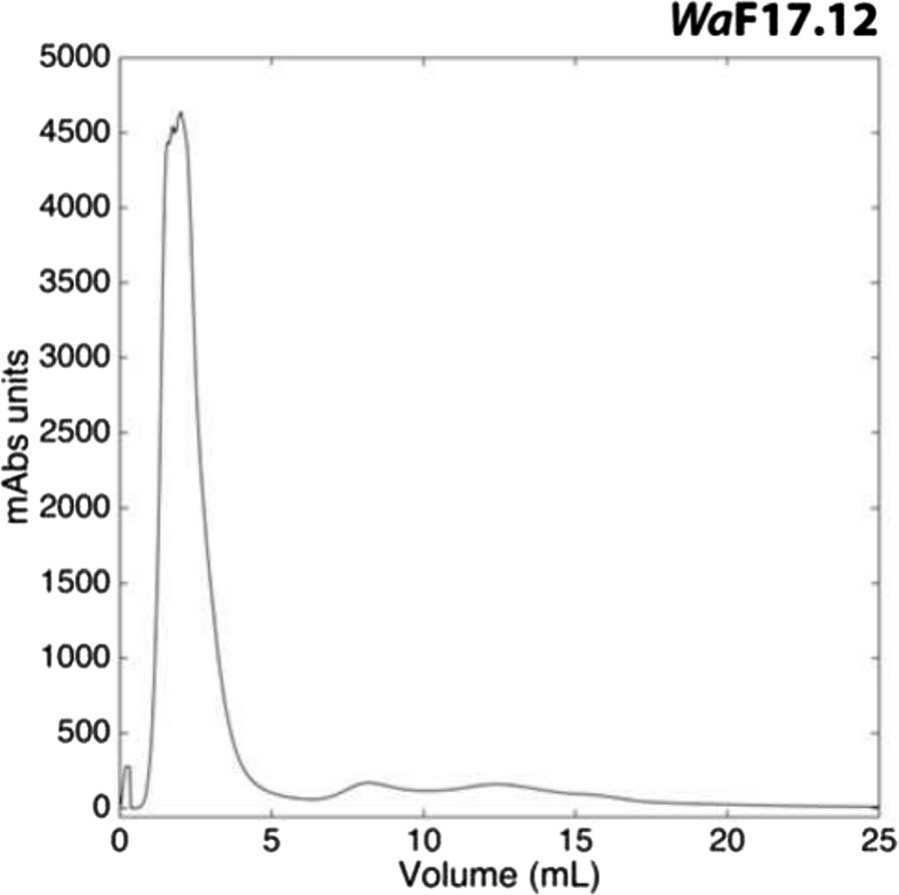
Anion-exchange chromatography profile. Profile obtained through the anion-exchange chromatography (DEAE) performed on the concentrated supernatant of the strain *Wa*F1712. The same elution profile was achieved also for the strains *Wa*ATCC 96603 (positive control) and *Wa*UM3 (negative control)

Three different strains of *W. anomalus* growing in conditions stimulating the production of toxin [14] were used: (1) the KT-producer *Wa*F17.12 isolated from *An. stephensi* mosquitoes; (2) the KT-producer *Wa*ATCC 96603 (positive control), and (3) the non KT-producer *Wa*UM3 (negative control). Anion-exchange chromatography analysis has revealed the presence of two major peaks in each yeast strains analysed: non-retained and retained fractions (Fig. 1). Due to similar chromatographic profiles, a killing activity test was performed in order to discriminate effective KT presence in the fractions obtained from the three different strains. Killing activity assay on the susceptible *Wa*UM3 strain, revealed KT presence only in the first eluted fraction of *Wa*F17.12 and *Wa*ATCC 96603 strains (see Additional file 1).

Accordingly, these fractions were used against *P. berghei* sporogonic stages.

### In vitro anti-plasmodial activity of *Wa*KTs

KTs purified from *Wa*F17.12 and *Wa*ATCC 96603 supernatants were tested on *P. berghei* cultures. KT concentrations were compatible with those of killer peptides used against the promastigotes of *Leishmania* sp., in vitro conditions [25]. The KTs secreted by both *W. anomalus* F17.12 and *W. anomalus* ATCC 96603 showed anti-plasmodial activity against the development of *P. berghei* sporogonic stages; whereas no effect was detected when the cultures were incubated with the purified supernatant of *Wa*UM3. Indeed, the ookinetes treated with non-retained fraction of *Wa*UM3 showed a comparable development with the control (see Additional file 2).

**Fig. 2:**
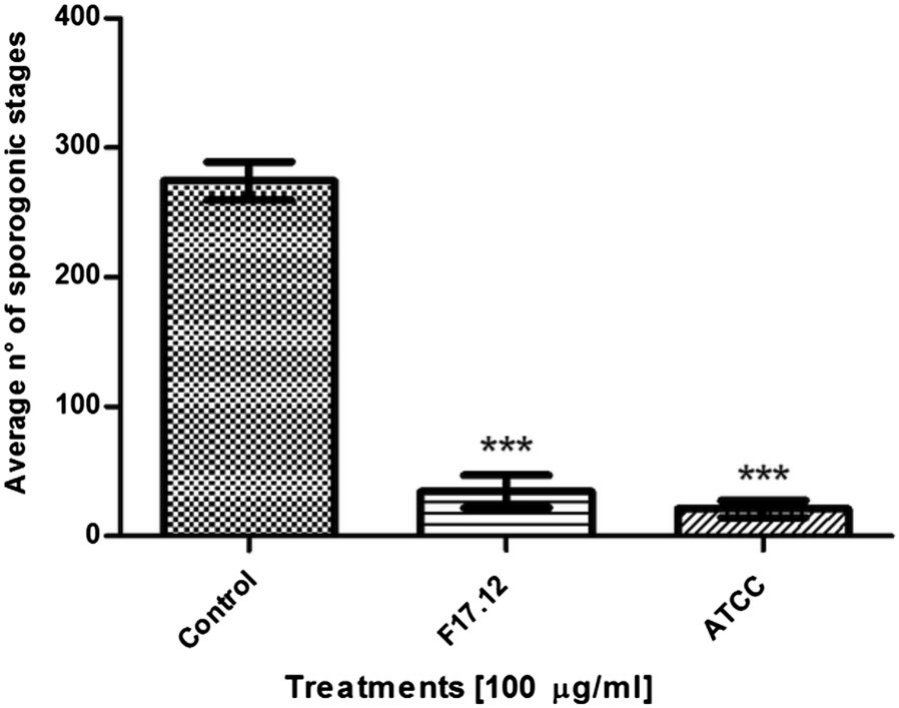
In vitro anti-plasmodial activity of *Wa*KTs against *Plasmodium berghei* sporogonic stages. The development of sporogonic stages showed inhibition rates of 87.5 and 92.3 % when they were incubated with 100 µg/ml of *Wa*F17.12 and *Wa*ATCC 96603 KTs, respectively, for 24 h at 19 °C. The histogram reports the average numbers of the sporogonic stages obtained from the cell count of three wells for each treatment and control (LC_50_ values were 61.3 and 64.6 µg/ml for *Wa*ATCC 96603 and *Wa*F17.12, respectively). The One Way ANOVA Bonferroni’s Multiple Comparison tests were used to perform the statistical analysis. Statistical significance is expressed as a p value. ***p < 0.001

**Fig. 3:**
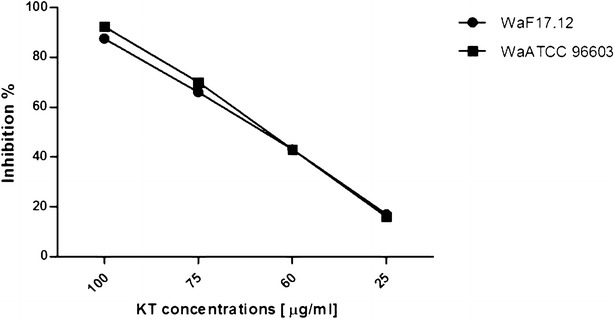
Inhibition percentages of *Plasmodium berghei* sporogonic stages development at different KTs concentration. The KTs of *Wa*F17.12 and *Wa*ATCC 96603 showed a dose-dependent activity at the tested concentrations of 100, 75, 60 and 25 µg/ml. Inhibition percentages upon exposure to 25, 60, 75 µg/ml of *Wa*F17.12 KT were approximately of 17, 43, 66 %, whereas for *Wa*ATCC 96603 KT were about 16, 48 and 70 %

Notably, KTs activity showed a dose-depending trend. Treatment with 100 µg/ml of *Wa*F17.12 and *Wa*ATCC 96603 KTs induced the highest inhibition percentages of the sporogonic stages development (87.5 and 92.3 %, respectively) (Fig. 2). Inhibition percentages upon exposure to 25, 60, 75 µg/ml of *Wa*F17.12 KT were approximately of 17, 43 and 66 %, whereas for *Wa*ATCC 96603 KT were about 16, 48 and 70 % (Fig. 3). The values of LC_50_ were 61.3 and 64.6 µg/ml for *Wa*ATCC 96603 and *Wa*F17.12, respectively, suggesting an equivalent activity of the two proteins.

### Investigation of morphological/structural alterations in treated parasites

**Fig. 4:**
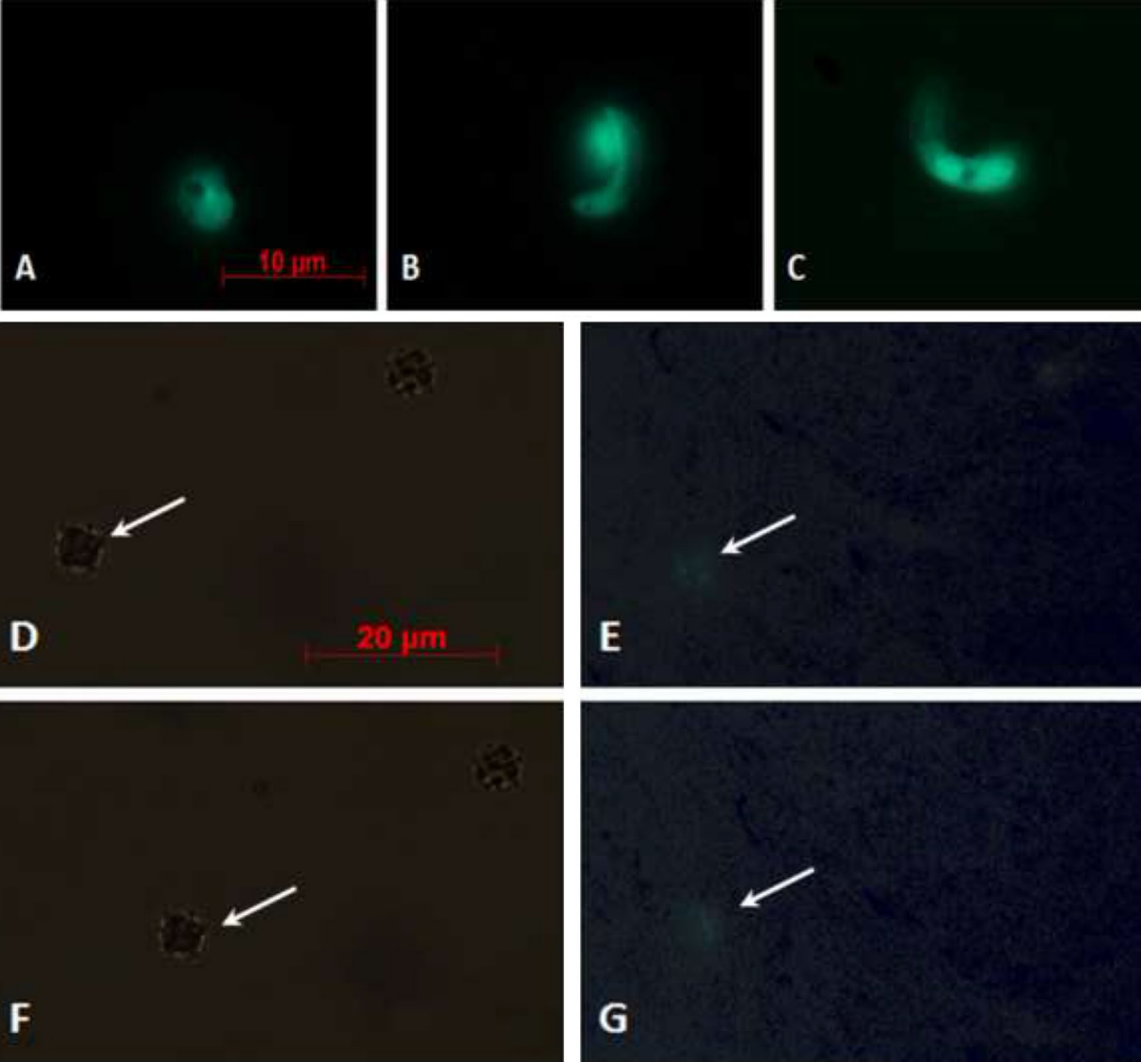
Fluorescence microscopy evaluation of GFP-transfected parasites. Different stages of untreated parasite development are shown in **a**–**c** (**a** zygote; **b** elongated zygote; **c** mature ookinete). **e** and **g** show faint fluorescent signals into zygotes (indicated by *white arrows*) treated with *Wa*F17.12-KT. In **d** and **f** phase-contrasts (the zygotes are indicated by *white arrows*) are shown. All the parasites were observed using a ×100 objective. In **a**–**c** *scale bar* 10 μm whereas in **d**–**g** *scale bar* 20 μm

**Fig. 5:**
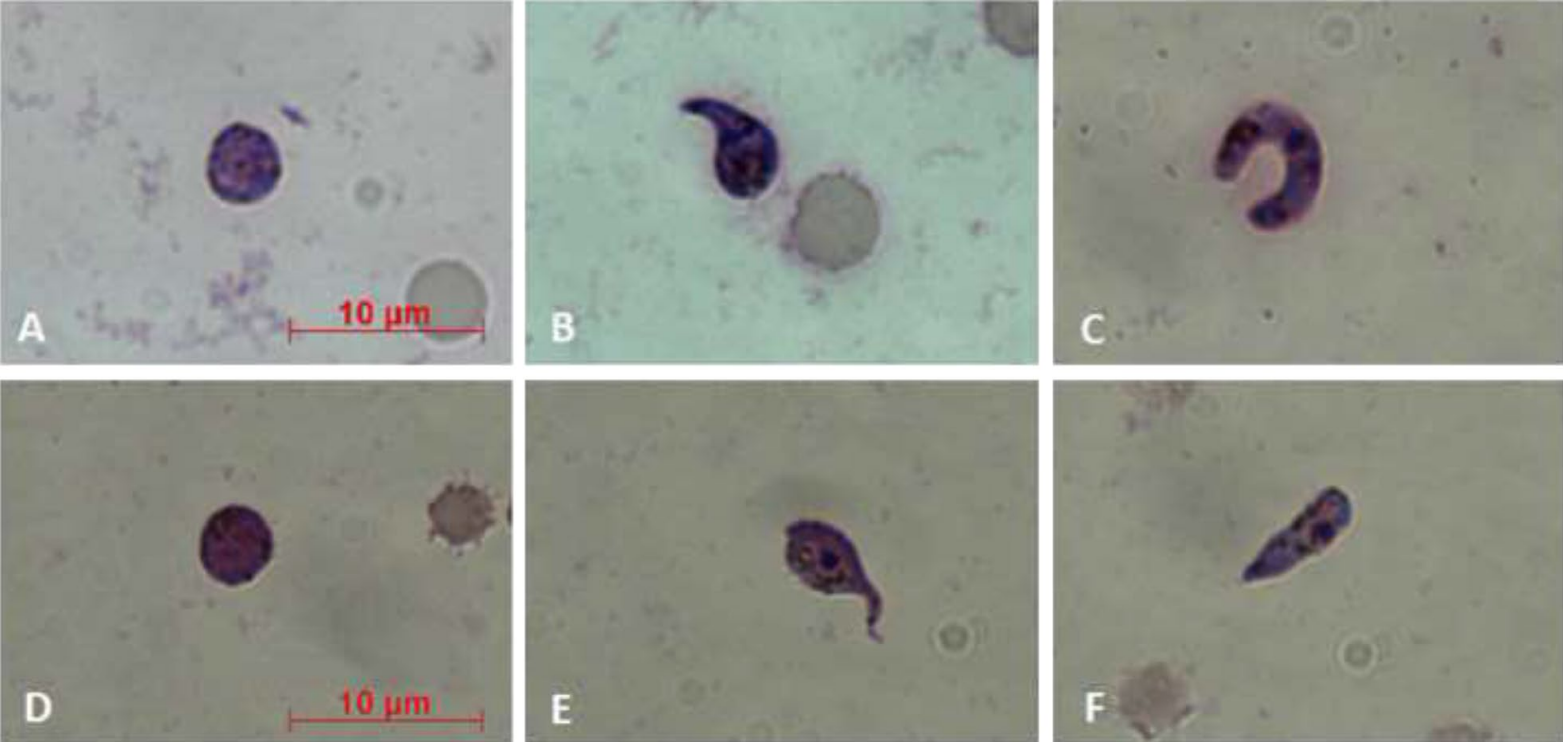
Images of *Plasmodium berghei* cultures in Giemsa-stained smears. Parasites sporogonic stages, both controls (**a**–**c**) and treated with 100 µg/ml of *Wa*F17.12 KT (**d**–**f**) were stained with Giemsa and analysed under light microscope (×100 objective). See text for details. *Scale bar* 10 μm

Upon GFP-parasites treatment with KTs, a lower fluorescence signal in the sporogonic stages was detected. Figure 4 shows a comparison between control and treated parasites from zygotes to ookinetes. Intracellular GFP leakage might result from KTs-induced alterations on the permeability of the parasites cell membrane. To better investigate KT-induced morphological changes in *P. berghei* sporogonic stages, smears of parasites cultures were stained with 10 % Giemsa. Morphologically, alterations of the post-zygotic stages were evidenced, whereas no particular differences were observed at the zygotes level (Fig. 5). In details, zygotes from both groups (controls and KTs-treated) appeared comparable in size with intensely colored cytoplasm while treated ones showed irregular borders and less defined cytoplasmic granules and crystalloid precursors (Fig. 5a, d). In control parasites, the ookinete development was characterized by a completed elongation process and the presence of a strong staining and well-defined crystalloid organelles surrounded with haemozoin (Fig. 5b, c). The morphological/structural alterations of the post-zygotic stages of the KT-treated parasites included: (1) irregular cell-shape and jagged cell borders; (2) a feeble staining of cytoplasmic region; (3) the lack of crystalloid assembly and (4) less-defined cytoplasmic granules, (Fig. 5e, f).

### Possible KT mechanism of action

The main antimicrobial mechanism of *W. anomalus* involves the direct killing of sensitive microorganisms by releasing of KTs that recognize specific cell-wall receptors on target membranes [27]. One of the proposed mechanism of action of these proteins is based on their interference with the glucans determining a β-1,3-glucanase activity [28].

To verify if the toxicity against the *P. berghei* sporogonic stages of KTs was mediated by β-glucanase activity, the KTs of both *Wa*F17.12 and *Wa*ATCC 96603 were treated with castanospermine, a β-glucanase inhibitor [26]. Castanospermine-induced inhibition of KTs was evaluated after 24 h incubation compared to controls (Table 1). Castanospermine induced a reduction in the killer activity of both proteins (from 79.7 to 46.2 % in the case of *Wa*F17.12 and from 88.5 to 49.5 % in the case of *Wa*ATCC 96603) confirming the possible correlation between the enzymatic activity of KTs and the killer phenotype of the yeast.

**Table 1:** Effects of castanospermine on KTs activity against *P. berghei*

Pretreatment	Treatment	Average number of sporogonic stages/well
β-Glucanase inhibitor (25 μM)	KTs (100 μg/ml)	
None	None	305 ± 22.7
None	*Wa*F17.12-KT	62 ± 11.5
None	*Wa*ATCC 96603-KT	35 ± 4.6
Castanospermine	*Wa*F17.12-KT	125 ± 8.1
Castanospermine	*Wa*ATCC 96603-KT	151 ± 4.5

## Discussion

The yeast *W. anomalus* is known to display natural antimicrobial properties against a wide range of microbes, including the protozoan parasites *Leishmania* sp. and *Acanthamoeba castellanii* [15, 25, 29]. Herein, through a rapid chromatographic protocol [30], *Wa*KT produced by *Wa*F17.12 strain, isolated from the major Asian malaria mosquito vector, *An. stephensi*, was purified. Furthermore, the first evidence of this KT inhibition potential against *P. berghei* sporogonic stages in laboratory cultures was provided. The obtained outcomes report an undeniable parasite inhibition up to 90 % compared to control samples. Alongside, although *Wa*ATCC 96603 reference strain has been already described for its robust multi-target killing activity, its efficacy against *P. berghei* sporogonic stages was additionally estimated.

Subsequently, *Wa*KT action mechanism was investigated through both enzymatic tests and Giemsa staining. Previous work demonstrated that KTs bind to cell-wall primary receptors, and then, move to secondary ones in the plasma membrane inducing the death of sensitive cells through DNA damages and apoptosis [25]. In particular, KT activity was associated with the hydrolysis of the β-glucans located in cell-wall membranes [28]. Notably, the β-1,3-glucans recognized by KT, are known to be present not only in fungi but also in cell-wall of parasites, as reported in the protozoa *Toxoplasma* and *Eimeria* [31], and they are implicated in the immune-protection against *P. berghei* infection in mice [32].

Moreover, it is reported that the bond between the KT and the membrane receptors causes the formation of transmembrane channels, determining the leak of intracellular materials [33]. A similar event was detected in amphotericin-treated promastigotes of *Leishmania major* suggesting a loss of intra-cellular material from apoptotic cells [25]. In this frame, a lower fluorescent signal in sporogonic stages treated with KT was observed respect to controls. The morphological and structural alterations observed in KT-treated parasites, using Giemsa staining, confirmed that *Wa*F17.12 KT targets the sporogonic stages of *Plasmodium*, interfering with the correct development of mature ookinetes.

Additionally, β-glucanase implication is also supported by the presence of the genes responsible for β-1-3-glucanase synthesis (*EXG*1 and *EXG*2) in the genome of *W. anomalus* [14, 34, 35]: their single or coupled silencing was in fact correlated with the lack of the yeast antimicrobial function; however, no direct relationship has been yet demonstrated between these genes and the KT activity [36].

For this reason, to evaluate the direct involvement of a β-glucanase activity of KT on malaria parasites, the β-glucanase inhibitor castanospermine was used. The inhibitory effect of castanospermine on the *Wa*KT activity confirmed a β-glucanase-mediated mechanism of action, resulting consistent with data on *Tetrapisispora phaffi* [26]. However it is worth to underline that inhibition percentages in castanospermine-treated samples is still lower with respect untreated control: this is easily explained by the shortage of a suitable molecule concentration to saturate all the KT active sites.

Finally, an in-depth analysis of our results reinforced the idea that KT, produced by the strain isolated from mosquitoes, interacts with specific receptors, the β-glucans, localized on the surface of *P. berghei* cells and strongly inhibits their development from gametocytes to ookinetes.

## Conclusion

The overall outcome from this study paves the way for better understanding of the effect of *Wa*KTs on the malaria parasite in the mosquito midgut, which coincides with the bottleneck in *Plasmodium* life cycle. Previously, Cappelli et al. showed that the *W. anomalus* strain associated to mosquito is able to produce a KT in *An. stephensi* [14]. Herein, it was demonstrated that this protein is strongly active against *P. berghei*, in vitro conditions. Future in vivo studies will focus on the action of KTs against rodent malaria parasites with the prospect to provide a natural tool for innovative malaria SC strategies.

## Additional files

10.1186/s12936-015-1059-7 Killing activity against *Wa*UM3 strain.
10.1186/s12936-015-1059-7 Ookinetes development under *Wa*UM3 non-retained fraction.

## Abbreviations

SC: Symbiotic control
KT: Killer toxin
GFP: Green fluorescent protein
CTRP: Circumsporozoite and TRAP related protein
IRBC: Infected red blood cells
YPD: Yeast extract peptone dextrose
MWCO: Molecular weight cut off
FPLC: Fast protein liquid chromatography
DEAE FF: Diethylaminoethanol fast flow
PBS: Phosphate buffered saline
LC_50_: Lethal concentration 50
