# Supplementary material for: A yeast strain associated to Anopheles mosquitoes produces a toxin able to kill malaria parasites
Source: Malar J. 2016 Jan 11;15:21. doi: 10.1186/s12936-015-1059-7 (PMC4709964; doi:10.1186/s12936-015-1059-7)

**Additional file 1: Killing activity against *Wa*UM3 strain**

10^5^ cells/ml of sensible strain *Wa*UM3 were treated with only PBS 1X (A) or the purified first eluted fraction of *Wa*UM3 (B), *Wa*F17.12 (C), *Wa*ATCC 96603 (D). The cell growth was evaluated after 12 h incubation at 26°C using a microscope and 40× objective (Carl Zeiss Axio Observer.Z1, Milan, Italy).


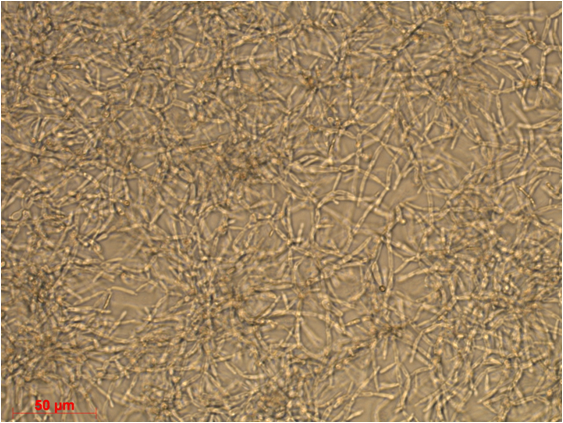

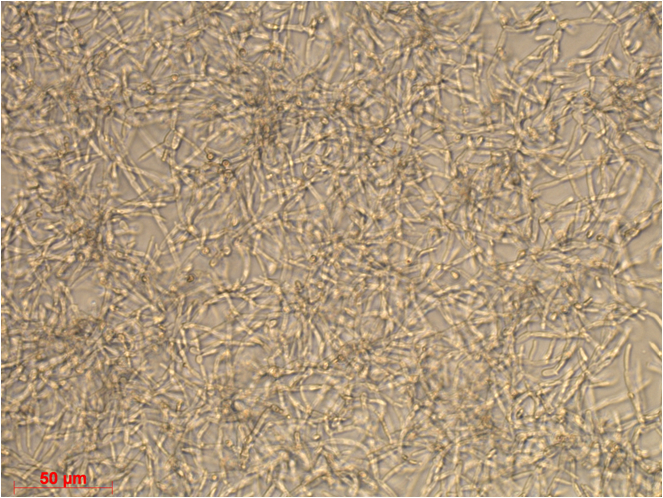

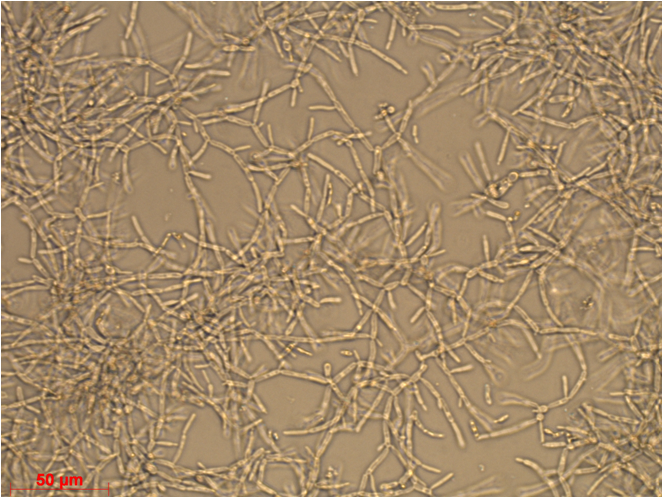

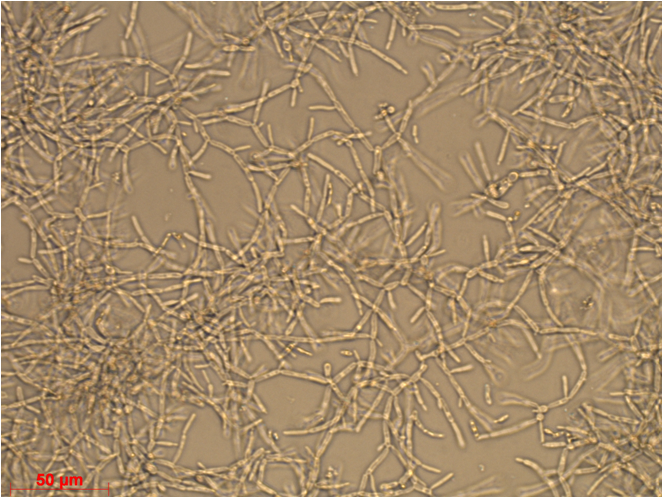


**A**

**D**

**B**

**C**

**A**


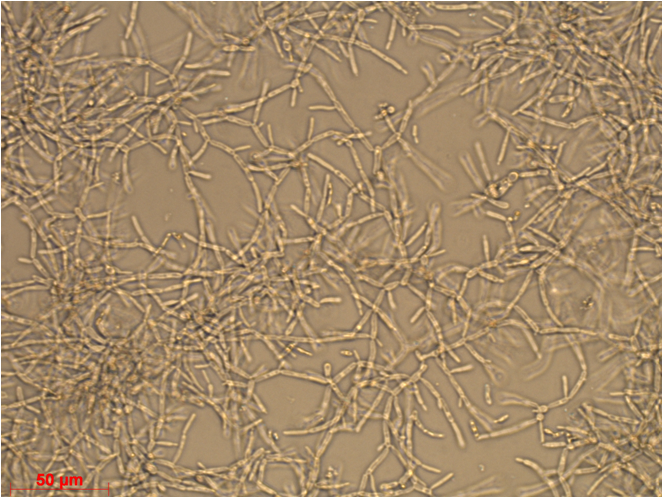

Supplement: Supplementary file 1 — 10.1186/s12936-015-1059-7 Killing activity against WaUM3 strain. [file 12936_2015_1059_MOESM1_ESM.docx]
