# Supplementary material for: A yeast strain associated to Anopheles mosquitoes produces a toxin able to kill malaria parasites
Source: Malar J. 2016 Jan 11;15:21. doi: 10.1186/s12936-015-1059-7 (PMC4709964; doi:10.1186/s12936-015-1059-7)

**Additional file 2: ookinetes development under *Wa*UM3 non-retained fraction.**

The sporogonic stages treated with non-retained fraction of *Wa*UM3 (A) and PBS 1× (B) show a comparable development after 24 h incubation at 19°C. The images were obtained using a fluorescent microscope and 40X objective (Carl Zeiss Axio Observer.Z1, Milan, Italy).


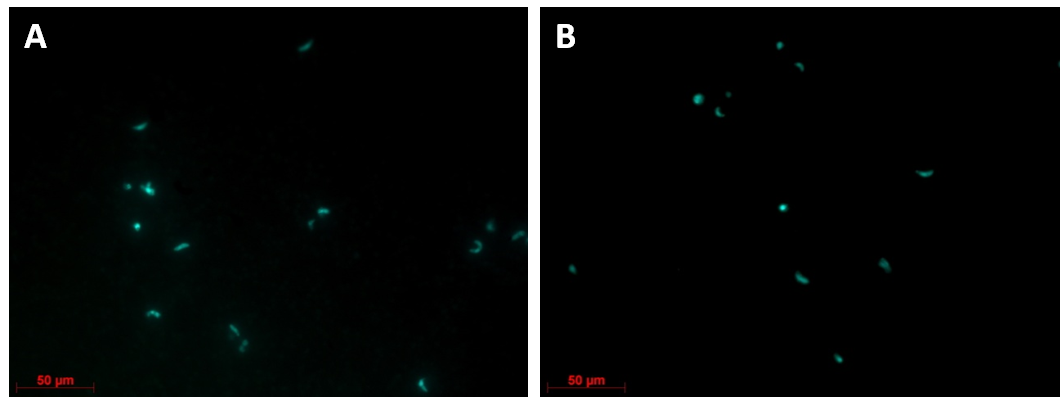

Supplement: Supplementary file 2 — 10.1186/s12936-015-1059-7 Ookinetes development under WaUM3 non-retained fraction. [file 12936_2015_1059_MOESM2_ESM.docx]
